# Cavka – a new automatic pharmacophore elucidation method in progress

**DOI:** 10.1186/1758-2946-3-S1-P31

**Published:** 2011-04-19

**Authors:** F Koelling, K Baumann

**Affiliations:** 1 Pharmaceutical Chemistry, University of Technology, 38106 Braunschweig, Germany

## 

Three dimensional pharmacophore models can be considered as an ensemble of steric and electronic features in space, which are necessary to ensure intermolecular interaction with a specific target in order to trigger or to block biological activity [1]. By identifying these features, a 3D pharmacophore model can be built in order to screen multi-conformatorial databases with the aim to detect compounds matching the pharmacophoric hypothesis and subsequently submit them to a biological testing. Even if a 3D crystal structure is at hand, the creation of a reliable pharmacophore model remains a challenging task.

CavKA (Cavity Knowledge Acceleration), our own in-house strategy employs the information of Co-crystallised ligand-receptor complexes for an automatic pharmacophore creation. Ligand features interacting with the binding site are detected and Grid [2] force field information is additionally taken into account as to weight and prioritize the identified features in question, to transform them into a pharmacophore model without any user intervention.

Our method is compared to LigandScout [3] and a custom MOE [4] implementation, similar to LigandScout, two powerful standard tools. Both are identifying ligand-receptor interactions to highlight important ligand features to be selected for creating pharmacophore models automatically. The performance is evaluated in a retrospective screening on the FieldScreen [5] dataset outlining strengths, weaknesses and as well as similarities of each method for the scrutinized targets.
